# Ketamine for depression relapse prevention following electroconvulsive therapy: protocol for a randomised pilot trial (the KEEP-WELL trial)

**DOI:** 10.1186/s40814-016-0080-0

**Published:** 2016-08-03

**Authors:** Martha Finnegan, Karen Ryan, Enda Shanahan, Andrew Harkin, Leslie Daly, Declan M. McLoughlin

**Affiliations:** 1 Department of Psychiatry and Trinity College Institute of Neuroscience, St. Patrick’s University Hospital, James’ St., Dublin 8, Ireland; 2 Trinity College Institute of Neuroscience, Trinity College Dublin, College Green, Dublin 2, Ireland; 3 School of Pharmacy and Pharmaceutical Sciences, Trinity College Dublin, College Green, Dublin 2, Ireland; 4 Centre for Training and Research in Analysis and Research, University College Dublin, Belfield, Dublin 4, Ireland

**Keywords:** Depression, Relapse prevention, Ketamine, Electroconvulsive therapy, Pilot trial

## Abstract

**Background:**

Major depressive disorder is a common debilitating illness that is the second leading contributor to the global burden of disease. Unfortunately, about 30 % of patients do not respond to adequate trials of antidepressants and/or psychotherapies. About 45–60 % of such treatment-resistant patients will remit with electroconvulsive therapy (ECT). However, relapse rates are high following ECT—38 % after 6 months. There is a need for better relapse prevention strategies. One possibility is to use ketamine, a competitive glutamate receptor antagonist used for anaesthesia. A recent paradigm shift in treating depression and understanding its biology has been the finding that ketamine has a robust, rapid-onset, though short-lived, antidepressant effect that is possibly mediated through neuroplastic effects. However, ketamine has not previously been reported on for relapse prevention.

**Methods/design:**

The main objective of this study is to conduct a randomised controlled pilot trial (*n* = 40) of a 4-week course of once-weekly ketamine infusions for relapse prevention following ECT for depression to assess trial procedures that will inform a future definitive trial. Participants with unipolar depression will be recruited prior to commencing ECT and be assessed weekly during the ECT course using the primary clinical outcome, the 24-item Hamilton Rating Scale for Depression (HRSD-24). Those who meet standard response criteria will be invited, on completing ECT, to be randomised in a 1:1 ratio to a course of four once-weekly infusions of ketamine or an active comparator midazolam, which mimics some of the effects of ketamine and may improve blinding over inactive placebo. Participants will be followed up over 6 months using the HRSD-24 to assess for relapse.

**Discussion:**

This is the first registered trial (NCT02414932, https://clinicaltrials.gov/ct2/show/NCT02414932) of ketamine for depression relapse prevention, an important possible use of this agent. The primary focus of the pilot trial is on feasibility. However, a 95 % confidence interval will be determined for the difference between ketamine and midazolam groups in 6-month relapse rates to help inform a future definitive trial.

**Trial registration:**

https://clinicaltrials.gov/ NCT02414932

Secondary Identifying numbers:

EudraCT number: 2014-000339-18

Sponsors’ Reference, Sponsor: St. Patrick’s Mental Health Services: 05/14

Research Ethics Committee Reference, Joint REC of St James’ and Tallaght Hospitals, Dublin: 2014-08-19

**Electronic supplementary material:**

The online version of this article (doi:10.1186/s40814-016-0080-0) contains supplementary material, which is available to authorized users.

## Background

### Depression, ECT and relapse

Major depressive disorder (MDD) is a debilitating mental illness with a lifetime prevalence of 12–20 % [1]. It is the most costly brain disorder in Europe, accounting for 1 % (€118 billion annually) of the total European economy [2]. Indeed, depression is currently the second largest cause globally for years lived with disability [3].

About 30 % of patients do not respond to antidepressants even after multiple trials with/without psychotherapies [4]. However, electroconvulsive therapy (ECT) offers up to 60 % of such treatment-resistant patients to complete remission [5–7]. ECT is a medically safe procedure and is more acutely effective than psychotherapy or antidepressants for severe, often treatment-resistant, depression [5]. The major concerns are cognitive side effects, but for most people, these are transient and many cognitive functions improve [8]. Treatments involve passing small electrical charges through the brain to induce a seizure lasting ~30 s under anaesthesia with a muscle relaxant. Six to 12 treatments are typically administered in a course, two to three times weekly [9]. Worldwide, 1.4 million people receive ECT annually, including nearly 260 people in Ireland [10].

In a recent meta-analysis, we found that taking antidepressants following successful ECT halves the risk for relapse (risk ratio = 0.49, *p* < 0.0001, NNT = 3.3) at 6 months from nearly 80 % [11], but mean relapse rates remain high: 27.1 % after 3 months and 37.7 % after 6 months [11]. Even continuation ECT (C-ECT), albeit mostly at non-adjustable fixed schedules, does not seem to improve 6-month relapse rates (37.2 %). Notably, these relapse rates are similar to those for patients who respond only after ≥3 antidepressant steps and most likely reflect the recurrent nature of treatment-resistant depression [4].

A major challenge now is how best to prevent relapse after successful treatment of depression with ECT. However, remarkably, the evidence base for relapse prevention in depression following any successful treatment is small. For example, the National Institute for Health and Care Excellence (NICE) in the UK have identified that evidence on relapse prevention in depression is limited and recommended research in this area. [12] To date, the reported randomised controlled trials for relapse prevention following successful antidepressant therapy have focused on the effect of 12 months’ tricyclic or SSRI antidepressant therapy, showing consistent but limited reduction in relapse rates [13]. As discussed in our review [11], there have been very few randomised, controlled trials focusing on relapse prevention after ECT. One study investigated the use of nortriptyline or nortriptyline and lithium for relapse prevention following successful ECT for depression [14]. Nortriptyline-lithium combination therapy had a marked advantage in time to relapse, superior to both placebo and nortriptyline alone. Other studies have focused on fixed-schedule continuation ECT, which has a relapse prevention effect in well-chosen groups [15].

### Ketamine as an antidepressant

Ketamine is a competitive glutamate *N*-methyl-d-aspartate receptor (NMDAR) antagonist with a half-life of 2–3 h. Ketamine has a remarkably rapid antidepressant effect, targeting core symptoms, in treatment-resistant depression when given as single sub-anaesthetic doses, usually a 40-min 0.5 mg/kg intravenous infusion [14]. Thereafter, robust antidepressant effects (~70 % responder rates) occur within 2–4 h and persist for a few days, i.e. beyond immediate NMDAR blockade [14]. These findings have led to the most exciting development in treating and understanding depression in over 50 years and represent a paradigm shift away from conventional slow-acting monaminergic antidepressants. Preclinical studies have shown that within just 2 h, ketamine increases synaptogenesis and spine formation in rodent prefrontal cortex and rapidly reverses chronic stress-induced depressive behaviours and prefrontal neuronal atrophy [15]. These effects are mediated, at least in part, via Akt/GSK-3/mammalian target of rapamycin (mTOR) signalling and increased dendritic translation of synaptic proteins [16], as well as deactivation of eukaryotic elongation factor 2 (eEF2) kinase, resulting in de-suppression of brain-derived neurotrophic factor (BDNF) translation [17]. BDNF mediates synaptic plasticity and is implicated in mechanisms of antidepressants and ECT [18]. Changes in blood mononuclear cell levels of phosphorylated mTOR, eEF2 and GSK-3beta have also been associated with response to ketamine [19], suggesting potential as biomarkers for response.

Ketamine is psychotomimetic, but at low dosage, it is safe, with patients and healthy controls occasionally experiencing mild dissociative and psychotic symptoms that resolve soon after finishing infusions [16–18]. To control for these effects, and also avoid “carry-over” effects in crossover studies while improving blinding, midazolam, at the sub-anaesthetic dose of 0.045 mg/kg, has been used as a control in parallel-group design trials rather than inactive placebo saline [19]. Ketamine can be a drug of abuse and chronic high-dose abuse can cause uropathy and dependency. However, repeated (e.g. 2–3/week for 2 weeks) infusions of sub-anaesthetic ketamine are safe with more sustained antidepressant effects [20, 21]. Two recent reviews of trials of ketamine for use as an antidepressant showed the most commonly used dosage is a 40-min infusion of 0.5 mg/kg [16, 22]. Bioavailability of ketamine is highest when administered intravenously [23]. In sub-anaesthetic doses, ketamine is a medically safe drug but can cause transient rises in pulse and blood pressure during infusion and for up to 80 min afterward. However, a recent review of ketamine in depression concluded that outside recreational usage, there have been no reports of persistent adverse effects with sub-anaesthetic uses of ketamine [24].

The effect of ketamine on cognition is unclear and has only been studied in acute treatment of depression [25]. There may be changes in visual and working memory [26] and an association between baseline neurocognitive performance and response to ketamine [27]. Optimum dosing and deliver of ketamine has not been established [25]. Ketamine has been used for ECT anaesthesia and is associated with earlier improvement and possibly fewer cognitive side effects but no overall better response [16, 20, 22, 28]. There have been insufficient studies of intramuscular, oral or intranasal ketamine for depression to currently warrant studying these preparations for relapse prevention [21]. While the half-life of ketamine is 3 h, in previous studies, the antidepressant effect was maintained for up to 2 weeks [29]. No trials have yet been reported, or registered, for using ketamine as an adjunctive treatment to reduce relapse rates following successful depression treatment—a potential use of ketamine that this trial will explore.

## Methods/design

### Study objective

The primary objective is to conduct a randomised, controlled, patient- and rater-blinded pilot study of ketamine vs. an active comparator (midazolam) for 4 weeks following successful ECT, to assess trial process to inform a future definitive trial.

### Secondary objective

To calculate a 95 % confidence interval for an unadjusted hazard ratio that will allow interpretation of statistical difference between ketamine and midazolam groups to assess ketamine for reducing 6-month relapse rates following successful ECT.

### Overview

This randomised, controlled pilot trial will take place over 30 months. The study will have an open recruitment phase (phase I) followed by a randomised treatment phase (phase II). We will initially recruit patients with unipolar major depressive disorder (Diagnostic and Statistical Manual of Mental Disorders, Fourth Edition (DSM-IV) criteria [30]) referred for ECT, who will be assessed weekly to identify those eligible to take part in the randomised controlled pilot trial in phase II. Participants who are successfully treated with ECT (phase 1) and continue to meet inclusion criteria will be randomly allocated in a 1:1 ratio to a 4-week course of either once-weekly ketamine at 0.5 mg/kg or the active comparator midazolam at 0.045 mg/kg (phase 2). The trial will take place under “real world” conditions with both groups continuing usual care (e.g. regular medications, psychological and other therapies and out-patient review) during the randomised treatment phase and thereafter. Participants will be followed up over 6 months following ECT to identify if and when relapse occurs.

### Site

This single-site study will take place in St. Patrick’s University Hospital, Dublin, an independent-sector 250-bed university teaching hospital that provides a national mental health service. About one third of all ECT in Ireland is administered at the centre [7, 31].

### Research ethics approval

Approval for this pilot trial was obtained from the joint authorised Research Ethics Committee of St. James’ and Tallaght Hospitals, Dublin. Site approval was also obtained from the relevant committee at St. Patrick’s University Hospital. Authorisation for the clinical trial was obtained from the Health Products Regulatory Authority of Ireland, the relevant body under the European framework for clinical trials, EudraCT (2014-000339-18). The study will be conducted in accordance with the principles that have their origin in the Declaration of Helsinki [32], in accordance with Good Clinical Practice (GCP), as defined by the International Conference on Harmonisation [33] (ICH), and in accordance with the ethical principles underlying European Union Directive 2001/20/EC and 2005/28/EC. The trial has been registered at clinicaltrials.gov (NCT02414932).

### Recruitment

In line with recommendations for pilot studies [34], a formal sample size calculation has not been performed. Twenty participants is an acceptable total number for the purposes of a pilot trial. For this pilot trial, we aim to recruit up to 20 patients per group, a total of 40. Response rates to ECT are 40–60 % [5], so at least 66 patients need to be initially recruited. Allowing for a 15 % drop-out rate, we will therefore seek to recruit 78 patients. We expect to recruit 78 participants within 16 months, 47 of whom will meet response criteria following ECT [5], and that 40 of these will additionally consent to be randomised and participate in the pilot trial.

### Consent

Written informed consent will be obtained by members of the research team using the study-specific consent form (Additional file 1). Potential participants will be provided with an information leaflet and letter of invitation (Additional file 1) and verbal information at the first point of contact with a member of the research team.

### Eligibility criteria

Participants will be current inpatients in university teaching hospitals in St. Patrick’s Mental Health Services, who have a diagnosis of unipolar MDD and are referred for ECT. Participants may be male or female, aged ≥18 years, and from a variety of geographical (within Ireland) and socioeconomic backgrounds. Participants will not have any medical condition that would preclude treatment with ECT or ketamine/midazolam.

To be eligible for inclusion in phase 1, each participant must meet each of the following criteria at screening and must continue to fulfil these criteria at baseline.

1. Subjects must be able and willing to give written informed consent and comply with the requirements of this study protocol.
2. Diagnosed with unipolar major depressive disorder (DSM-IV), have a 24-item Hamilton Rating Scale for Depression (HRSD-24) of ≥21 and be referred for ECT.
3. Female subjects of child-bearing potential and male subjects whose partner is of child-bearing potential must be willing to ensure that they or their partner use effective contraception during the randomised treatment phase (phase II) and for 5 weeks thereafter.

Subjects are excluded from the study if any of the following criteria are met at screening:

1. Allergy/sensitivity to study medications or their ingredients.
2. Subjects who have participated in another study and received any other investigational agent within 6 months.
3. Any condition rendering patient medically unfit for ECT; general anaesthesia; ketamine or midazolam—assessed by physical examination, routine haematology and biochemistry investigations prior to enrolment.
4. Medications that may significantly alter the pharmacokinetics of ketamine (e.g. ketoconazole, clarithromycin) are contraindicated during the trial, and participants taking any of these medications at screening will be excluded from the trial.
5. Subjects who have a history of drug or alcohol use that, in the opinion of the investigator, would interfere with adherence to study requirements.
6. Known history of, or documented positive hepatitis B or C or HIV infection, advanced malignancy or terminal illness.
7. Scheduled for non-trial procedures requiring general anaesthesia during the study.
8. Active suicidal intention.
9. Dementia, intellectual disability or a score on the standardised Mini Mental State Examination (sMMSE) of <24.
10. Lifetime history of bipolar affective disorder.
11. Current history of post-traumatic stress disorder.
12. Other axis I diagnosis (DSM-IV).
13. ECT in the 6 months prior to recruitment.
14. Currently a prisoner or residing in a nursing home.

For inclusion in the randomised controlled trial (phase 2), following successful ECT, patients must additionally have:

1. Received a significant course of ECT (i.e. at least five sessions)
2. Achieved at least response criteria (i.e. ≥60 % decrease from baseline HRSD-24 score and score ≤16 on two consecutive weekly ratings)
3. Have a nominated adult who can stay with them for 24-h on out-patient treatment days
4. sMMSE [35] score of ≥24

### Assessments

The primary clinical outcome measure is the relapse rate at 6 months as measured using the objectively rated HRSD-24 [36]. To enter the study, patients must score ≥21. Subjective mood ratings will be also measured using the Quick Inventory of Depressive Symptoms, self-report version (QIDS-SR) [37]. Baseline assessment will also include diagnosis and treatment history: diagnosis of major depressive disorder will be confirmed using the mood episodes module of the Structured Clinical Interview for DSM-IV Axis I Disorders (SCID) [38]. The Maudsley Staging Method for Treatment Resistant Depression (MSTRD) [39] will be used to provide a measure of treatment-resistance. Handedness will be recorded with the Edinburgh Handedness Questionnaire (EHQ) [40]. The National Adult Reading Test (NART) [41] will measure premorbid intelligence.

Additional baseline data from patient interview and case-note review will include age, sex, weight, height, occupation, educational attainment, duration of index depressive episode, number of previous depressive episodes, previous ECT, history of medical illness and surgical treatments, personal and family history of alcohol/substance dependency, presence of psychotic symptoms (detected by SCID) and current medications and other therapies. Changes in medications will be documented at follow-up interviews (Table 1, schedule of events).

**Table 1:** Schedule of enrolment, assessments and interventions

	Phase 1: ECT patients and healthy controls			Phase 2: ECT responders randomised to ketamine or midazolam			
Assessment	Baseline (pre-ECT)	Weekly during ECT course	End of ECT course	Pre-infusions	Infusions 1–4; weeks 1–4	Follow-ups: weeks 6, 8, 12 and 20	Final follow-up week 26
Diagnosis and treatment							
Background, SCID, NART, CTQ	✓						
Treatment review	✓		✓		✓ (1–4)	✓ (6–20)	✓
Clinical outcomes							
HDRS-24	✓	✓	✓		✓✓✓✓ (1–4)	✓ (6–20)	✓
QIDS-SR	✓	✓	✓		✓✓✓✓ (1–4)	✓ (6–20)	✓
Cognitive outcomes							
ACE-R	✓		✓		✓4th		✓
Digit spans	✓		✓		✓4th		✓
Trails A + B	✓		✓		✓4th		✓
sMMSE	✓		✓		✓4th		✓
AMI	✓		✓		✓4th		✓
Ketamine effects							
CADSS					✓✓✓✓ (1–4)		
BPRS					✓✓✓✓ (1–4)		
YMRS					✓✓✓✓ (1–4)		
PRISE					✓✓✓✓ (1–4)		
Consent							
Signed consent	✓			✓			
Verbal assent	✓	✓	✓		✓✓✓✓ (1–4)	✓ (6–20)	✓
Eligibility							
Eligibility check	✓	✓	✓	✓	✓✓✓✓ (1–4)	✓ (6–20)	✓
Randomisation							
Allocation				✓			

Participants will be assessed weekly during ECT using the HRSD-24 and QIDS-SR. Response to ECT is defined as achieving ≥60 % decrease from baseline HRSD-24 and score ≤16 on two consecutive weekly ratings. Remission criteria are ≥60 % decrease in HRSD from baseline and score ≤10 on two consecutive weekly ratings. Those identified as being ECT responders will be invited to participate in the two-group parallel-design randomised controlled pilot trial. The advantages of randomisation and blinding in this group will also be used to perform studies of peripheral blood biomarkers as potential predictors of response to ketamine (Additional file 2). During infusion sessions in the randomised treatment phase, HRSD-24 scores will be obtained 60 min before the infusion begins and at +120 and +240 min afterwards. Baseline scores on sleep and appetite items will be maintained for repeated measures within 1 day. The +240 min HRSD-24 scores will serve as the weekly post-ECT scores up to follow-up week 4.

Ketamine psychotomimetic effects and adverse events will be assessed using the following instruments before, during (+35–40 min) and after (+240 min) infusions of ketamine or midazolam:

- Clinician-Administered Dissociative States Scale (CADSS) [42]
- Brief Psychiatric Rating Scale (BPRS; four-item positive symptom subscale) [43]
- Young Mania Rating Scale (YMRS; mood item) [44]
- Patient-Rated Inventory of Side Effects (PRISE) [45]

Participants will be followed up for 6 months following ECT with repeated questionnaires comprising treatment review plus HRSD-24 and QIDS-SR at weeks 6, 8, 12 and 20 post-ECT. Criteria for relapse are ≥10 point increase in HRSD-24 compared to baseline phase 2 score plus HRSD ≥16; in addition, increase in the HRSD should be maintained 1 week later (if indicated, additional follow-ups will be arranged). Hospital admission, further ECT and deliberate self-harm/suicide also constitute relapse. Timing of these events will be recorded. A final follow-up session in week 26 will comprise HRSD-24, QIDS-SR and cognitive outcomes.

There are no published data on effects of ketamine on cognition in ECT responders. We will use the following battery pre- and post-ECT course, after the fourth infusion and at 6 -month follow-up. The post-ECT assessment will serve as baseline for the randomised pilot trial. Where appropriate, parallel versions will be used to reduce practice effects. Global cognition will be assessed with the sMMSE [35]. Immediate short-term memory, attention and working memory will be measured using Forward and Backward Digit Spans [46]. Motor and psychomotor speed will be assessed using the Trail Making Test (part A) [46]. Frontal-executive function will be rated by Trail Making Test (part B) [46] plus letter and category verbal fluencies [47]. Anterograde verbal memory will be tested using the verbal learning component of the Addenbrooke’s Cognitive Examination III (delayed and immediate recall of a seven-item address) [48]. Retrograde amnesia for autobiographical information will be measured using the Kopelman Autobiographical Memory Interview (K-AMI) [49].

### Interventions

Participants in phase I will receive ECT and usual care and will be monitored weekly using the HRSD-24 for response. ECT will be administered twice-weekly with hand-held electrodes according to Royal College of Psychiatrists’ guidelines and as previously described [7, 50]. Briefly, the Mecta 5000 M device (Mecta Corporation, USA) will be used and seizure duration measured by EEG monitoring. Methohexitone (0.75–1.0 mg/kg) will be used for anaesthesia with suxamethonium (0.5–1.0 mg/kg) as muscle relaxant. Brief-pulse (1.0-ms pulse width; current amplitude 800 mA) ECT will be administered twice weekly (Mecta 5000 M device, Mecta Corp., Portland, Ore.; maximum 1200 mC), using methohexitone (0.75–1.0 mg/kg) anaesthesia and succinylcholine (0.5–1.0 mg/kg) for muscle relaxation (16, 22). Seizure threshold (ST) will be established by a method of limits, as previously described [7], at the first session, and subsequent treatments will be given at 1.5 × ST for BL ECT and 6.0 × ST for RUL ECT. Stimulus charge will be titrated upward as required during treatment courses following a standard stimulus dosing protocol. To reflect routine clinical practice, number of ECT treatments will be determined by referring physicians who will be blind to randomisation. ECT characteristics will be recorded.

Participants who have successfully responded to ECT in phase I and meet inclusion criteria to continue to the randomised controlled pilot trial in Phase II will be randomised (1:1) to receive four once-weekly infusions of either ketamine or midazolam. The regimen of four weekly infusions was chosen to facilitate subjects travelling for appointments and because ketamine has proven effects as a rapid-acting antidepressant but has not yet been studied as a series of infusions for relapse prevention. Patients and raters will be blind to treatment. The first infusion will be administered within 2 weeks of completing ECT and may be administered as an inpatient or outpatient; further infusions will take place as an outpatient. Each infusion will take 40 min, and monitoring will take place for 200 min post-commencement of infusion. Ketamine hydrochloride 10 mg/ml infusion at 0.5 mg/kg (Pfizer Healthcare Ireland) or midazolam hydrochloride (Hypnovel) 10 mg/5 ml solution at 0.045 mg/kg (Roche Products Ireland Ltd) will be made up as 50 ml colourless saline solutions and administered intravenously via an infusion pump. This dose and administration was chosen based on a previous randomised controlled trial of ketamine and midazolam in which these dosages were well-tolerated [51].

During each treatment session, participants will be monitored for heart rate, blood pressure, pulse oximetry and electrocardiogram changes. Adverse or psychotomimetic effects of either agent will be monitored using the CADSS, BPRS (four-item positive symptom subscale), YMRS (mood item) and the PRISE administered before, during and after infusions. Cognitive outcomes will be repeated in week 4. All other assessment measures will be repeated weekly, including treatment review, HRSD-24 and QIDS-SR.

Participants will be advised not to drive or operate heavy machinery for 24 h post-commencement of infusions and be provided with information on recent changes to the Road Traffic Act (Ireland) 2014, which includes provisions for roadside intoxication testing. Participants will be asked to ensure they have a nominated adult who can stay with them for 24 h on outpatient treatment days and will be contacted by a researcher 24 h after each session to enquire about side effects.

Treatment-as-usual will continue during the trial. Participants will continue to receive pharmacotherapy, psychotherapy or other therapeutic inputs as recommended by their treating team for the duration of the trial. There are no provisions for post-trial care or improving adherence due to the pilot trial design. Premature termination of the trial may take place in the event of new information regarding safety of investigative medicinal products becoming available, unsatisfactory progression of the trial, major breach of data confidentiality or if in the participants’ best interests. Subjects have the right to voluntarily discontinue study treatment or withdraw from the study at any time for any reason without any consequences. Subjects must discontinue the investigational medicinal product(s) and be withdrawn from the study for any of the following reasons:

- Withdrawal of consent by the subject
- Any medical condition that the investigator or sponsor determines may jeopardize the subject’s safety if she or he continues receiving the study treatment
- Pregnancy
- Ineligibility (either arising during the study or retrospectively having been overlooked at screening)
- An adverse event which requires discontinuation of the study medication
- Treatment failure and disease progression
- Lack of compliance with the study and/or study procedures (e.g. dosing instructions, study visits)
- Loss to follow-up—at least three documented attempts must be made to contact any subject lost to follow-up.

### Outcomes

The focus of this study is on trial process with assessment of the primary clinical outcome being secondary. However, efficacy data will be collected in the course of the trial and will be reported as part of the study findings.

Process outcomes that will help to inform a future definitive ketamine relapse prevention trial include information on the following:

1. Recruitment methods and rate
2. Willingness of participants to be randomised
3. Willingness of participants to complete assessments
4. Randomisation
5. success of blinding of participants and raters
6. Ability to administer a course of four weekly ketamine infusions
7. Medical safety and acceptability of ketamine infusions in an ECT responder population
8. Rates of adverse dissociative and psychiatric events
9. Adherence to allocated treatment
10. Adherence to follow-up
11. Reasons for drop-out from treatment
12. Reasons for drop-out from follow-up
13. A 95 % confidence interval for the difference between the ketamine and midazolam groups in 6-month relapse rates to help inform a future definitive trial

The primary outcome relating to efficacy (the assessment of which is not a primary objective) is the relapse rate at 6 months as measured by HRSD-24. Subjective mood rating as measured by scores on QIDS-SR is a secondary efficacy outcome. The following safety evaluations will be performed during the study: adverse event monitoring, vital signs, cognitive and clinical assessments. Safety endpoints are:

(i) Tolerability of ketamine vs. midazolam in terms of cognitive outcomes
(ii) Tolerability of ketamine vs. midazolam in terms of psychotomimetic effects as measured by scores on CADSS, BPRS, YMRS and PRISE
(iii) Number of adverse effects in ketamine vs. midazolam groups

**Fig 1:**
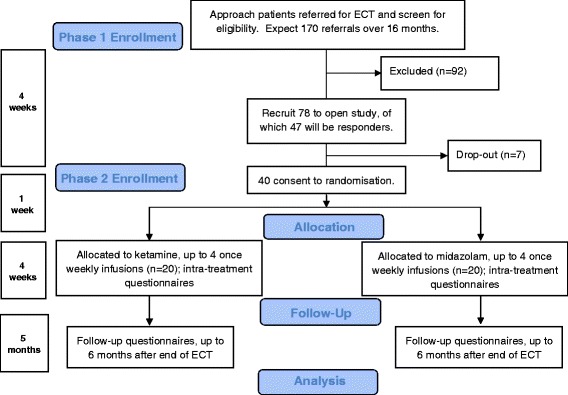
Consort diagram

This trial has been designed and will be reported in line with the Consolidated Standards of Reporting Trials (CONSORT) guidelines [52] (Fig. 1).

### Allocation sequence generation and concealment

ECT responders will be randomised after post-ECT assessment. Subjects will be randomly assigned to one of two treatment groups in a 1:1 ratio. Computerised random allocation, using randomly permuted blocks, will be done independently by the Centre for Support and Training in Analysis and Research (CSTAR, University College Dublin, www.cstar.ie). Allocation information will be provided by means of a randomisation list prepared by CSTAR, available only to the anaesthetist to ensure allocation concealment. Researchers involved in ratings will not have access to information regarding treatment allocation.

### Blinding and unblinding

To ensure patient safety during infusions and in the post-infusion period, the anaesthetist administering the ketamine/midazolam infusions will not be blinded but he will not be involved in assessments or data analysis. Patients, raters and the trial statistician will remain blinded. Success of blinding for patients and raters will be assessed after the first treatment. A set of envelopes containing allocation information will remain unopened but may be used where emergency unblinding is indicated. Unblinding for one or all participants will take place if it is in the best interests of the participants.

### Statistical methods

Pilot trial data will be analysed on an intention-to-treat basis for all participants who complete at least one infusion and one post-infusion evaluation. Data analyses will be performed blinded to allocation by the trial statistician. As this is a pilot trial and small numbers of participants are involved, no missing values will be imputed. Data will be analysed using IBM PASW (SPSS) version 22 and “R” (R Foundation for Statistical Computing, Austria).

Demographic and baseline data will be summarised for each treatment group by presenting descriptive statistics. Descriptive statistics will also be used to report: rates of recruitment, willingness to be randomised, willingness to complete assessments, medical/cognitive/psychotomimetic/general adverse events between groups, adherence to allocated treatments, adherence to follow-up between groups and reasons for drop-outs between groups.

Relapse-free survival times will be compared between groups using Kaplan-Meier survival curves and log-rank test. As this is a pilot trial and insufficiently powered to achieve statistical significance, there will be no formal comparison of the two treatment groups in the pilot trial. However, Cox proportional hazard regression analysis will provide a 95 % confidence interval for an unadjusted hazard ratio for a future definitive trial.

As this is a pilot trial and small numbers of participants are involved, no missing values will be imputed. As per the pilot trial design, the primary outcomes are those relating to reasons for dropout and these will be presented using descriptive statistics.

### Data management

A trial-specific operating procedure for data quality assurance will be followed by all researchers and involves eight levels of quality assurance. Researchers will be trained in administration of the primary assessment tool used in this study, the HRSD-24, and training will be repeated every 6 months to ensure good inter-rater reliability. The study will comply with the Data Protection (Amendment) Act, 2003, Ireland. All documents will be stored safely in a designated locked filing cabinet in a locked office within the Research Building at St. Patrick’s University Hospital, and confidentiality will be observed at all times. With the exception of the informed consent form, subjects will be referred to only by their subject identification number on all study-specific documents, whether hard copies or electronic. Data analysis will take place in another facility (CSTAR, University College Dublin), and data will be anonymised prior to secure transfer to CSTAR for analysis.

### Trial management

The Trial Steering Committee, chaired by an independent clinician and including a service user representative, will meet on a six-monthly basis. The Trial Management Group, comprising trial researchers, will meet on a weekly basis. A Data Monitoring Committee (DMC), chaired by an independent clinical researcher and comprising an independent biostatistician and independent trial methodologist, will meet every 6 months to review blinded data and reports prepared by the trial statistician. The trial results will be published and communicated to participants, and authorship eligibility guidelines of the International Committee of Medical Journal Editors (ICJME) will be followed. There is no intended use of professional authors. Important amendments to the trial protocol will be communicated to all relevant agencies. The sponsor owns the final dataset; access will be managed through the Principal Investigator. There are no contractual agreements limiting access to data for investigators. There are no plans to grant public access to the participant-level dataset or statistical code. Trial processes will not be independently audited due to the pilot trial design.

## Discussion

This is the first registered trial investigating the potential use of ketamine for relapse prevention in depression and the first study to investigate intravenous ketamine for relapse prevention following successful response to ECT. Only one other registered study proposes to investigate this issue; however, that study uses intranasal esketamine (NCT02493868). Previous studies have shown that ketamine has a rapid effect in acute depression, maintained up to 2 weeks, but repeated infusions have not been studied in a recovered population for the purpose of relapse prevention. The focus of this pilot trial is on process outcomes to help inform a future definitive trial. Strengths of the study include its double-blind design and use of independent randomisation. Notwithstanding the potential utility of the trial in assessing the safety and practicability of this treatment protocol, there are some limitations. The small number of proposed participants in this pilot trial (up to *n* = 40) limits the statistical analyses which can be confidently applied to the ones described here. The nationwide catchment area of the trial site may pose difficulties in retention of participants from phase I to phase II due to travel challenges. Treatment-as-usual will continue for all participants during the pilot trial, resulting in a heterogeneous participant group. This pragmatic design will, however, improve generalisability of the results and applicability to a future definitive trial.

### Trial status

Recruitment commenced

## Abbreviations

Version III; BPRS, Brief Psychiatric Rating Scale; CADSS, Clinician-Administered Dissociative States Scale; CSTAR, Centre for Support and Training in Analysis and Research, University College Dublin; CTQ, Childhood Trauma Questionnaire; DSM-IV, Diagnostic and Statistical Manual of Mental Disorders, Fourth Edition; ECT, electroconvulsive therapy; EHQ, Edinburgh Handedness Questionnaire; HRSD-24, Hamilton Depression Rating Scale, 24-item version; K-AMI, Kopelman Autobiographical Memory Interview; MDD, major depressive disorder; MSTRD, Maudsley Staging for Treatment-Resistant Depression; NART, National Adult Reading Test; NMDA, *N*-methyl-d-aspartate; PRISE, Patient-Rated Inventory of Side Effects; QIDS-SR, Quick Inventory of Depressive Symptoms, Self-Report, 16-item; SCID, Structured Clinical Interview for DSM-IV Axis I Disorders; sMMSE, Standardised Mini Mental State Exam; YMRS, Young Mania Rating Scale

## Additional files

Additional file 1: Informed consent materials. (DOCX 26 kb)
Additional file 2: Details of biological studies. (DOCX 25 kb)
