# Supplementary material for: Ketamine for depression relapse prevention following electroconvulsive therapy: protocol for a randomised pilot trial (the KEEP-WELL trial)
Source: Pilot Feasibility Stud. 2016 Aug 3;2:38. doi: 10.1186/s40814-016-0080-0 (PMC5153900; doi:10.1186/s40814-016-0080-0)
Supplement: Additional file 1: — Informed consent materials. (DOCX 26 kb) [file 40814_2016_80_MOESM1_ESM.docx]

**Consent Form Phase I**

**The KEEP WELL Study**

**Ketamine for depression relapse prevention following ECT: a randomised pilot trial with blood biomarker evaluation**

**EudraCT No: 2014-000339-18**

**Please contact Prof. Declan McLoughlin on ext 3385 for more information**

The participant must complete this form herself/himself

**PLEASE TICK YOUR RESPONSE IN THE APPROPRIATE BOX**

- I have read and understood the attached Participant Information Leaflet….. Yes □ No □⁪
- I have had the opportunity to ask questions and discuss the study ………… Yes □ No □
- I have received enough information about this study ……………………… Yes □ No □
- I understand that I am free to withdraw from the study at any time without

giving a reason and without this affecting my future medical care ..……….. Yes □ No □

- I agree to provide blood cells and plasma for analysis of protein expression.... Yes □ No □
- I agree to provide blood cells and plasma for the analysis of DNA and RNA,

including messenger RNA and micro RNA ........................................................Yes □ No □

- I understand that confidential anonymous data from my participation in the trial must be

retained securely by the research team for at least five years and will not be retained for

more than ten years.. . . . . . . . . . . . . . . . . . . . . . . . . . . . . . . . . . . . . . . . . . . . . . Yes □ No □

- I agree to take part in this study of my own free will and without prejudice

to my legal/ethical rights. . . . . . . . . . . . . . . . . . . . .. . . . . . . . . . . . . . . . . . . . . Yes □ No □

Participant’s Signature: _________________________ Date: ___________________

Participant’s Name in Print: _________________________ Date: ___________________

Witness Signature: * _________________________ Date: ___________________

Witness’ Name in Print: _______________________________________

Investigator’s Signature: _________________________ Date: ___________________

Investigator’s Name in Print: _______________________________________

**Please attach the Participant Information Sheet to this Consent Form, ask the participant to sign and date it and, where appropriate, place a copy of both in the participant’s case notes.**

***Witness must be someone other than the Investigator**

**Consent Form Phase II**

**The KEEP WELL Study**

The participant must complete this form herself/himself

**PLEASE TICK YOUR RESPONSE IN THE APPROPRIATE BOX**

- I have had the opportunity to ask questions about Phase II of this study …… Yes □ No □
- I have received enough information about Phase II of this study …………… Yes □ No □
- If I am invited to participate in Phase II, I agree to be randomly allocated to one

of two treatment groups and I understand that I may be allocated to a placebo

treatment group...................................................................................................Yes □ No □⁪

- I agree to receive four infusions of either ketamine or midazolam . . . . . . . . . . Yes □ No □
- I understand that I am free to withdraw from the study at any time without

giving a reason and without this affecting my future medical care ..……….. Yes □ No □

- I agree to provide blood cells and plasma for analysis of protein expression....Yes □ No □
- I agree to provide blood cells and plasma for the analysis of DNA and RNA,

including messenger RNA and micro RNA .......................................................Yes □ No □

- I understand that confidential anonymous data from my participation in the trial must be

retained securely by the research team for at least five years and will not be retained for

more than ten years.. . . . . . . . . . . . . . . . . . . . . . . . . . . . . . . . . . . . . . . . . . . . . . Yes □ No □

- I agree to take part in this study of my own free will and without prejudice

to my legal/ethical rights. . . . . . . . . . . . . . . . . . . . .. . . . . . . . . . . . . . . . . . . . Yes □ No □

Participant’s Signature: _________________________ Date:

Participant’s Name in Print: _________________________

Witness Signature: * _________________________ Date:

Witness’ Name in Print: _________________________

Investigator’s Signature: _________________________ Date:

Investigator’s Name in Print: _________________________

**Please attach the Participant Information Sheet to this Consent Form, ask the participant to sign and date it and, where appropriate, place a copy of both in the participant’s case notes**
