# Supplementary material for: Ketamine for depression relapse prevention following electroconvulsive therapy: protocol for a randomised pilot trial (the KEEP-WELL trial)
Source: Pilot Feasibility Stud. 2016 Aug 3;2:38. doi: 10.1186/s40814-016-0080-0 (PMC5153900; doi:10.1186/s40814-016-0080-0)
Supplement: Additional file 2: — Details of biological studies. (DOCX 25 kb) [file 40814_2016_80_MOESM2_ESM.docx]

**The Keep well trial**

**Appendix: Biological Studies**

**Background to Biological Studies**

Identifying a simple peripheral blood-based biomarker for diagnosis of depression and treatment response would be a major step. The most appropriate biomarkers for initial evaluation are neuroplasticity markers, i.e. BDNF[1-3] as well as phosphorylated species of mTOR, eEF2 and GSK-3beta[4-6], all of which have been associated with ketamine’s antidepressant effect. Several meta–analyses have established that both serum and plasma BDNF are reduced in major depression and rise to normal levels with effective antidepressant treatment[7].

The **objective** for the biological studies which will take place as part of this pilot trial is to evaluate changes in blood levels of neuroplasticity-associated proteins as biomarkers to predict the effect of ketamine in six-month relapse rates in ECT responders.

**Methods**

Consented ECT responders who participate in the randomised controlled trial of four infusions of ketamine or midazolam at sub anaesthetic doses will be assessed for changes in blood levels of neuroplasticity-associated proteins during the first ketamine infusion. Blood samples for plasma will be collected at four time points before, during and after the first ketamine/midazolam infusion (-60, +40, +120, and +240 minutes) and changes in biomarkers including BDNF, pMTOR, pGSK3beta and peEF2 will be compared between ketamine and midazolam groups. Six-month relapse rates (assessed as described in main text) between groups will be compared with changes in biomarkers to assess these proteins as predictors of the effect of ketamine on relapse rates in ECT responders.

Relapse will be assessed using HRDS-24. Criteria for relapse are ≥10 point increase in HRSD-24 compared to baseline Phase II score plus HRSD ≥16; in addition, increase in the HRSD should be maintained one week later (if indicated, additional follow-ups will be arranged). Hospital admission, further ECT, and deliberate self-harm/suicide also constitute relapse. Timing of these events will be recorded.

At the first infusion session, changes in blood levels of BDNF, pMTOR, pGSK3beta and peEF2 in response to the ketamine/midazolam will be compared at time points -60, +40, +120 and +240 minutes. Blood samples for plasma will be collected using EDTA vacutainer tubes and centrifuged to generate plasma that will be aliquoted and stored at -80^o^C. BDNF levels will be determined using ELISA (ChemiKine, USA) following the manufacturer’s instructions. PBMCs will be collected, pelleted and stored at -80^o^C as described above. Semi-quantitative immunoblotting of PBMC lysates will be used to measure changes in levels of the other ketamine-induced proteins using antibodies against activated mTOR phosphorylated at serine residue 2448 (AbCam, USA), inhibitory serine-9 phosphorylated GSK-3beta (Cell Signalling Technology, USA), and phospho-eEF2 (Thr56; Cell Signalling Technology, USA) with appropriate secondary antibodies and controlling respectively for total levels of mTOR, GSK-3beta and eEF2 measured using relevant antibodies. Protein bands will be identified using standard chemiluminescent techniques (Millipore) visualised in a darkbox imager (LAS 3000, Fujifilm) and analysed using ImageJ (Image Processing and Analysis in Java) software.

**Data analyses** The sample size is that required for a pilot study and can thus be considered a convenience sample for the purposes of these biological studies. Changes in the various protein levels will be compared between the two groups over the different time points by ANCOVA, using family history of alcohol dependency, body mass index and pre-ketamine HRSD-24 as covariates. Data will also be analysed with linear models to test relationships between changes in protein levels and relapse over the six-month follow-up as well as cognitive outcomes. Where relevant, age, gender, psychosis and level of treatment-resistance in the index depressive episode, remission status, and NART will be used as covariates.

1. Duman RS, Li NX: **A neurotrophic hypothesis of depression: role of synaptogenesis in the actions of NMDA receptor antagonists**. *Philosophical Transactions of the Royal Society B-Biological Sciences* 2012, **367**(1601):2475-2484.

2. Autry AE, Adachi M, Nosyreva E, Na ES, Los MF, Cheng PF, Kavalali ET, Monteggia LM: **NMDA receptor blockade at rest triggers rapid behavioural antidepressant responses**. *Nature* 2011, **475**(7354):91-U109.

3. Haile CN, Murrough JW, Iosifescu DV, Chang LC, Al Jurdi RK, Foulkes A, Iqbal S, Mahoney JJ, De La Garza R, Charney DS *et al*: **Plasma brain derived neurotrophic factor (BDNF) and response to ketamine in treatment-resistant depression**. *International Journal of Neuropsychopharmacology* 2013.

4. Li NX, Lee B, Liu RJ, Banasr M, Dwyer JM, Iwata M, Li XY, Aghajanian G, Duman RS: **mTOR-Dependent Synapse Formation Underlies the Rapid Antidepressant Effects of NMDA Antagonists**. *Science* 2010, **329**(5994):959-964.

5. Li NX, Liu RJ, Dwyer JM, Banasr M, Lee B, Son H, Li XY, Aghajanian G, Duman RS: **Glutamate N-methyl-D-aspartate Receptor Antagonists Rapidly Reverse Behavioral and Synaptic Deficits Caused by Chronic Stress Exposure**. *Biological Psychiatry* 2011, **69**(8):754-761.

6. Yang C, Zhou ZQ, Gao ZQ, Shi JY, Yang JJ: **Acute Increases in Plasma Mammalian Target of Rapamycin, Glycogen Synthase Kinase-3 beta, and Eukaryotic Elongation Factor 2 Phosphorylation After Ketamine Treatment in Three Depressed Patients**. *Biological Psychiatry* 2013, **73**(12):E35-E36.

7. Brunoni AR, Lopes M, Fregni F: **A systematic review and meta-analysis of clinical studies on major depression and BDNF levels: implications for the role of neuroplasticity in depression**. *International Journal of Neuropsychopharmacology* 2008, **11**(8):1169-1180.
